# Editorial: Rehabilitation robotics: challenges in design, control, and real applications, volume II

**DOI:** 10.3389/fnbot.2024.1437717

**Published:** 2024-06-04

**Authors:** Francisco Romero-Sánchez, Luciano Luporini Menegaldo, Josep M. Font-Llagunes, Massimo Sartori

**Affiliations:** ^1^Departamento de Ingeniería Mecánica, Energética y de los Materiales, Escuela de Ingenierías Industriales, Universidad de Extremadura, Badajoz, Spain; ^2^Programa de Engenharia Biomédica, PEB/COPPE, Universidade Federal do Rio de Janeiro (UFRJ), Rio de Janeiro, Brazil; ^3^Biomechanical Engineering Lab, Department of Mechanical Engineering and Research Centre for Biomedical Engineering, Universitat Politécnica de Catalunya, Barcelona, Spain; ^4^Health Technologies and Innovation, Institut de Recerca Sant Joan de Déu, Barcelona, Spain; ^5^Department of Biomechanical Engineering, University of Twente, Enschede, Netherlands

**Keywords:** wearable robots, exoskeletons, exosuits, rehabilitation, human augmentation, neuromusculoskeletal modeling, assistive devices

## Introduction

Rehabilitation Robotics is a relatively new field of study that requires a combination of knowledge and skills across multiple disciplines, ranging from mechanical design methodology and control systems theory to clinical neurosciences. The landscape of rehabilitation robotics has undergone a transformative shift, moving from rigid, clinical-centric solutions to more adaptable and user-centric approaches. The transition from conventional, hard-bodied systems to soft, lightweight, and fully wearable devices has been fueled by insights from biological systems. Furthermore, this evolution has been characterized by the accommodation of the individual user needs in terms of ergonomics, as well as the demands of their specific disabilities and rehabilitation protocols.

In the First Volume of this Frontiers Research Topic advances in the transition from conventional, hard-bodied systems to soft, lightweight, and fully wearable devices were addressed. The Second Volume of the Research Topic continues to explore that transition, presenting novel approaches to integrate biologically inspired actuators to liberate rehabilitation devices from the limitations of rigid supports, thereby improving user comfort and mobility.

Research in this field is currently focused on different topics. For example, regarding the design of the devices, novel designs of rehabilitation devices maintain flexibility and adaptability of the human skeletal system by using exomuscles to produce functional movements. Functional electrical stimulation during actuation has also been proven to aid in recovering neuroplasticity.

Moreover, synergistic principles have revolutionized the design of rehabilitation actuators, simultaneously enhancing their control and efficiency. The total number of actuators has also been reduced by combining actuators of different natures. By harnessing the natural synergies within the human body, researchers have achieved more streamlined and intuitive device interfaces, facilitating a smoother and more natural interaction between the user and the device.

Advancements in modeling and simulation techniques have also played a pivotal role in the evolution of rehabilitation robotics. By leveraging sophisticated computational models, researchers can now accurately assess and compensate for user fatigue, thereby prolonging the efficacy and usability of rehabilitation devices. Furthermore, these simulation tools enable researchers to simulate real-world scenarios, providing valuable insights into the potential benefits and challenges of using assistive devices in everyday settings.

Collectively, these research advancements have initiated in a new era of portable rehabilitation devices, providing unprecedented levels of versatility, comfort, and effectiveness. In this Frontiers Research Topic, we showcase a diverse array of novel techniques for the design, simulation, sensing, and control of rehabilitation devices, including powered exoskeletons, neuroprostheses, and equipment designed to seamlessly integrate rehabilitation into daily living environments beyond the confines of traditional clinical settings.

The second Volume of the Research Topic, “Rehabilitation Robotics: Challenges in Design, Control, and Real Applications, vol.II” provides an overview of this research area through six excellent contributions, covering various fundamental and complementary aspects within the field of Rehabilitation Robotics. Of the six articles, three focus on lower limb exoskeletons. Notably, each article addresses a different stage of biorobotic device development. Shi et al. present the development of a highly innovative reconfigurable exoskeleton, detailing its progression from conceptual design to pilot testing on healthy subjects. In contrast, the article by Lau and Mombaur uses the TWIN (Vassallo et al., 2020) exoskeleton, an advanced development stage device, to tackle the challenging problem of sit-to-stand motion. The authors employ optimal control to determine torque profiles for an “assisted as needed” actuation. Lastly, using logistic regression analysis, Taki et al. conducted a retrospective cohort study on the highly developed commercial product Hybrid Assistive Limb (HAL). Among the other contributions, Ratz et al. propose an exquisite, minimalist, portable, minimally-actuated haptic hand, and forearm trainer, describing the entire human-centered design process. Finally, two articles address topics strongly related to reinforcement learning control, a highly current topic, focusing on gait synthesis (Su and Gutierrez-Farewik) and force control in human-robot manipulator interaction (Xiao et al.).

## Challenges in design, control, and real applications

A simple object in most rehabilitation routines involving gait is still an anchor to obtain a natural motion. While crutches offer safety and stability, they also introduce an unnatural motion requiring additional coordination effort. Lau and Mombaur provide a comprehensive investigation into the use of crutch-less exoskeletons, focusing mainly on one of the most challenging motions, especially for the elderly population: sit-to-stand. Their study delves into the dynamics of sit-to-stand motions with default exoskeleton configurations involving crutches. Motion capture and optimal control methods are used to evaluate and compare the crutch-less sit-to-stand dynamics with the exoskeleton. The findings underscore the potential of lower-limb exoskeletons to assist frailer older adults in performing sit-to-stand transitions. Nevertheless, none of the devices in the market are currently suitable for this population, and the mandatory use of crutches imposes health challenges on them. Future recommendations to make the motion suitable for geriatric users include fine-tuning the weights of objective functions and considering the alterations in blood flow within the geriatric cardiovascular system. This could be achieved through sophisticated modeling techniques or by incorporating blood flow dynamics into the objective function. Such refinements promise to enhance the usability and effectiveness of exoskeleton-assisted sit-to-stand movements for elderly individuals undergoing rehabilitation.

The sit-to-stand challenges may also be part of the design process. Shi et al. contribute to this Research Topic by introducing a pioneering reconfigurable behavioral assistive robot that seamlessly merges the functionalities of an exoskeleton robot and an assistive standing wheelchair, facilitated by an innovative mechanism based on a four-bar linkage. This versatile device assists with walking, standing up, supported standing, and wheelchair mobility. In their study, Shi et al. model the sit-to-stand motion to characterize the functional capacity of the joints and, consequently, select the appropriate device actuators. The exoskeleton design encompasses both an exoskeleton module and a conformal transformation module, optimizing the assisted sitting function and ensuring wheelchair comfort. The researchers found a notable reduction in the primary lower limb muscles' demands by measuring electromyographic signals and plantar pressure. Despite some limitations, the challenge of sit-to-stand is addressed directly through the design of the exoskeleton itself, showcasing the interdisciplinary approach employed in tackling complex mobility issues.

Regarding gait synthesis and compensation strategies, recent studies have proposed control models with high-dimensional inputs and outputs that can reproduce human movement. Still, most of them do not account for human physiology. Su and Gutierrez-Farewick propose a reinforcement learning algorithm and a musculoskeletal model including trunk, pelvis, and leg segments to develop control modes that drive the model to walk. The simulations were achieved without reference motion capture data. Results show stable gait patterns at the prescribed walking velocities, leading to realistic human gait simulations. Moreover, this model can be applied to study optimal phenomena in walking with or without muscle weakness. Although individualized muscle parameters, accurate reproduction of internal and external forces, and other factors that affect how a subject interacts with the external environment may be required to improve the simulations, the work presented here constitutes an advancement for the accurate and individualized representation of pathological gait.

Physical prototypes also need compensation strategies to reflect a proper interaction between the rehabilitation device and the subject. The work of Xiao et al. analyzes robot-skin interaction scenarios. The robot end-effector has a probe that touches the skin and follows a predetermined trajectory. A sensor between the robot and the probe collects the force signal generated during this interaction. A force controller is required to regulate the contact state, ensuring the robot adheres to the reference force. A reference force is also established to ensure safety during the contact process. Xiao et al. present a robot force controller based on the Gaussian Mixture Model/Gaussian Mixture Regression (GMM/GMR) algorithm. The initial force control strategy is established using impedance control, augmented by integrating reinforcement learning with traditional control strategies. The proposed algorithm incorporates online and offline compensation strategies, enhancing its robustness and versatility to adapt to different skin environments. This method improves the controller's adaptability and reliability, ensuring safe and consistent interaction between the robot and human skin. The proposed approach is particularly beneficial for applications requiring delicate and precise robot-skin interactions, such as in medical and caregiving robots, where maintaining appropriate contact force is critical for safety and effectiveness.

Lastly, in this Research Topic, the human is placed at the center of rehabilitation device design. Rätz et al. built and evaluated a portable, cost-effective hand training mechanism for stroke rehabilitation that addresses both motor and sensory deficits, enabling increased training dosage and continued therapy at home. The study evolved a minimally-actuated hand training device for stroke rehabilitation into a safe, aesthetic, and functional prototype suitable for minimally supervised or unsupervised use. The system is also cost-effective and provides meaningful haptic feedback. Usability testing with healthy participants, including therapists, indicated that the device was easy to set up and intuitive, with realistic haptic feedback and generally positive user experiences. The work highlights the importance of continuous testing and stakeholder involvement in development.

Human-centered design is also relevant to personalizing treatment, especially for gait rehabilitation devices. Taki et al. investigate the effectiveness of robot-assisted gait training, particularly using the Hybrid Assistive Limb (HAL) to promote walking independence of post-stroke individuals. The study aimed to identify characteristics of stroke patients who may not benefit from gait training with HAL. Through a retrospective cohort study involving 82 stroke survivors, factors such as age, severity of paralysis (Brunnstrom recovery stage), and timing of HAL initiation were examined as predictors of walking independence. Logistic regression analysis was utilized to assess the impact of these factors. The study concluded that older age, greater paralysis severity, and delayed initiation of HAL-assisted training were associated with an increased likelihood of walking dependence upon hospital discharge.

## Conclusions and future perspective

This Research Topic provided a comprehensive and complementary overview of the most critical aspects of developing rehabilitation robots. Readers will find excellent guidelines, illustrated through real-life examples, for conducting clinically oriented research on rehabilitation robots, encompassing the entire process from conceptual design to mature technology assessment in large patient cohorts. All papers emphasize fundamental aspects, ranging from technical considerations related to advanced control algorithms and system dynamics, to the human-centered design and usability of affordable rehabilitation systems. Innovative devices and biomechanical methods, such as those shown in this Research Topic, will significantly impact the lives of an increasing elderly population and individuals with disabilities, underscoring the benefits of using personalized, versatile, and user-centric rehabilitation solutions to meet diverse needs.

The collaboration among engineers, physical therapists and patients is paramount in shaping the design landscape of rehabilitation devices, particularly neuroprostheses and rehabilitation robots tailored to the patients' needs. This multidisciplinary approach underscores the considerable effort required to develop clinically efficient solutions seamlessly integrated into rehabilitation routines.

Looking ahead, the insights gleaned from this Research Topic indicate a growing use of biomechanical models and simulation techniques to aid in the design and control of rehabilitation devices. By leveraging these tools, researchers can optimize device design and explore human-device interactions through virtual testing in different scenarios and device configurations. This approach streamlines the design process and offers invaluable insights into device functionality and user experience.

Furthermore, refining control strategies and advancing data acquisition and processing techniques may further imporve rehabilitation outcomes. By orchestrating precise and timely actuation, these technologies promise to enhance the efficacy of rehabilitation interventions, empowering patients to achieve their therapeutic goals more efficiently.

The ongoing work to minimize actuators' dimensions and energy requirements holds the promise of unlocking the potential for portable rehabilitation devices. This development will extend the reach of rehabilitation beyond clinical settings, facilitating more extensive and immersive rehabilitation experiences during daily living in domestic and community environments.

Lastly, integrating biosignal feedback and the ability to interface directly with the neuromusculoskeletal system, exemplified by functional electrical stimulation and spinal cord stimulation, heralds a new era in rehabilitation device design. These advancements offer unprecedented opportunities to address the complex challenges inherent in rehabilitating individuals with motor disabilities, paving the way for more personalized and effective interventions tailored to individual patient needs.

## Author contributions

FR-S: Writing – review & editing, Writing – original draft. LM: Writing – review & editing. JF-L: Writing – review & editing. MS: Writing – review & editing.
